# Glypican-3 targeted positron emission tomography detects sub-centimeter tumors in a xenograft model of hepatocellular carcinoma

**DOI:** 10.1186/s13550-023-00980-9

**Published:** 2023-04-27

**Authors:** Kevin P. Labadie, Adrienne L. Lehnert, Aimee L. Kenoyer, Donald K. Hamlin, Andrew D. Ludwig, Alan F. Utria, Sara K. Daniel, Tara N. Mihailovic, Alexander Prossnitz, Johnnie J. Orozco, Yawen Li, D. Scott Wilbur, Robert S. Miyaoka, James O. Park

**Affiliations:** 1 Department of Surgery, University of Washington School of Medicine, 1959 NE Pacific Street, Health Sciences Bldg. Room BB-442, Box 356410, Seattle, WA 98195-6410 USA; 2 Department of Radiology, University of Washington School of Medicine, 1959 NE Pacific Street, Seattle, WA 98195 USA; 3 Clinical Research Division, Fred Hutch Cancer Research Center, 100 Fairview Ave N, Seattle, WA 98109 USA; 4 Department of Radiation Oncology, University of Washington School of Medicine, 616 NE Northlake Pl., Seattle, WA 98105 USA; 5 Department of Bioengineering, University of Washington, 3720 15th Ave NE, Seattle, WA 98195 USA

**Keywords:** Hepatocellular carcinoma (HCC), Glypican-3 (GPC3), Immuno-positron emission tomography (PET), Targeted molecular therapy

## Abstract

**Background:**

Early intrahepatic recurrence is common after surgical resection of hepatocellular carcinoma (HCC) and leads to increased morbidity and mortality. Insensitive and nonspecific diagnostic imaging contributes to EIR and results in missed treatment opportunities. In addition, novel modalities are needed to identify targets amenable for targeted molecular therapy. In this study, we evaluated a zirconium-89 radiolabeled glypican-3 (GPC3) targeting antibody conjugate (^89^Zr-αGPC3) for use in positron emission tomography (PET) for detection of small, GPC3^+^ HCC in an orthotopic murine model. Athymic nu/J mice received hepG2, a GPC3^+^ human HCC cell line, into the hepatic subcapsular space. Tumor-bearing mice were imaged by PET/computerized tomography (CT) 4 days after tail vein injection of ^89^Zr-αGPC3. Livers were then excised for the tumors to be identified, measured, bisected, and then serially sectioned at 500 μm increments. Sensitivity and specificity of PET/CT for ^89^Zr-αGPC3-avid tumors were assessed using tumor confirmation on histologic sections as the gold standard.

**Results:**

In tumor-bearing mice, ^89^Zr-αGPC3 avidly accumulated in the tumor within four hours of injection with ongoing accumulation over time. There was minimal off-target deposition and rapid bloodstream clearance. Thirty-eight of 43 animals had an identifiable tumor on histologic analysis. ^89^Zr-αGPC3 immuno-PET detected all 38 histologically confirmed tumors with a sensitivity of 100%, with the smallest tumor detected measuring 330 μm in diameter. Tumor-to-liver ratios of ^89^Zr-αGPC3 uptake were high, creating excellent spatial resolution for ease of tumor detection on PET/CT. Two of five tumors that were observed on PET/CT were not identified on histologic analysis, yielding a specificity of 60%.

**Conclusions:**

^89^Zr-αGPC3 avidly accumulated in GPC3^+^ tumors with minimal off-target sequestration. ^89^Zr-αGPC3 immuno-PET yielded a sensitivity of 100% and detected sub-millimeter tumors. This technology may improve diagnostic sensitivity of small HCC and select GPC3^+^ tumors for targeted therapy. Human trials are warranted to assess its impact.

**Supplementary Information:**

The online version contains supplementary material available at 10.1186/s13550-023-00980-9.

## Background

Hepatocellular carcinoma (HCC) is the most common primary malignancy of the liver with an increasing incidence worldwide [1]. Surgical resection in select patients is effective and can achieve a 5-year overall survival of 70% [2]. Early tumor recurrence in the liver, or early intrahepatic recurrence (EIR), occurs in up to 50% of patients and is associated with reduction in survival [3–5]. EIR is multifactorial and can be secondary to undetected early cancer during initial diagnosis [6, 7]. Conventional imaging modalities, such as multiphase magnetic resonance imaging (MRI) and computerized tomography (CT) utilizing the LIRADS system, boast high sensitivity for HCC greater than 1 cm in size, but are less sensitive and specific for smaller lesions [8, 9]. Therefore, new diagnostic modalities are needed.

Receptor-targeted molecular technologies have demonstrated promise for cancer imaging and therapy [10]. Immuno-positron emission tomography (immuno-PET) combines targeting agents with positron emitting radioisotopes to detect targets associated with malignant and benign diseases [11]. Detection of these targets may improve identification of lesions and selection of patients for targeted therapies. Immuno-PET is being developed for HCC against several tumor-associated antigens including CD146, CD38 and glypican-3 (GPC3) [12–17]. Glypican-3 is an antigen expressed on the majority of HCC and minimally expressed in normal liver, making it a promising antigen for targeted technologies [18, 19].

Our group developed a GPC3 targeting PET probe using zirconium-89 (Zr^89^-aGPC3), and we hypothesize it will identify small GPC3^+^ tumors, which we test in an orthotopic xenograft model of HCC.

## Methods

### Orthotopic, cell-line xenograft model development

The University of Washington Institutional Animal Care and Use Committee reviewed and approved all protocols (UW IACUC Protocol #4304-02). All applicable institutional and/or national guidelines for the care and use of animals were followed and we carried out procedures in compliance with the ARRIVE guidelines. We purchased 8-week-old female athymic Nu/J mice (Jackson Laboratories, Stock No: 002019) and housed them in accordance with the University of Washington Office of Animal Welfare guidelines for the humane use of animals. HepG2-Red-FLuc (HepG2) cells expressing GPC-3 and *Luciferase* from PerkinElmer (Bioware, cat. no. BW134280, RRID:CVCL_5I98) were cultured, suspended in Matrigel (BD Biosciences) and injected into the subcapsular hepatic space. Detailed information regarding orthotopic model development is included in the Additional file 1: Supplementary Methods.

### Histopathologic processing

We harvested livers after PET imaging for histologic analysis. The livers were bisected at the site of tumor injection, embedded and serially sectioned at 500 μm intervals. A whole-slide scanner (Hamamatsu Nanozoomer) imaged each liver section and measured the diameter of the tumor. More detailed process is included in Additional file 1: Supplementary Methods.

### Production of ^89^Zr***-***DFO-***α***GPC3 and.^89^Zr***-***DFO-***α***BHV1

We generated αGPC3 IgG1-producing hybridomas through the Fred Hutchinson Cancer Research Center antibody core facility as previously described [13]. Conjugation of αGPC3 and BHV1 (Bovine Herpes Virus 1), a non-targeting irrelevant isotype-matched antibody, with deferoxamine (DFO) and radiolabeling with ^89^Zr is described in Additional file 1: Supplementary Methods*.*

### Biodistribution studies

To assess biodistribution of ^89^Zr-αGPC3, tumor-bearing mice were euthanized at 4 h, 1 d or 7 d after tail vein injection and tumors and normal organs were harvested and weighed. ^89^Zr activity was assayed with a gamma counter. The percent injected dose of radioisotope per gram (% ID/g) of blood, tumor, or organ was calculated after correcting for radioactive decay using an aliquot of the injectate and these values were used to calculate tumor-to-normal organ ratios of absorbed radioactivity.

### Blood clearance studies

After tumor-bearing mice were injected with ^89^Zr-αGPC3 or ^89^Zr-αBHV, blood was sampled via serial retro-orbital blood at 5, 15, 30, 60, 120, 240 min, and then on necropsy at 1 d and 7 d after injection. We measured radioactivity of blood samples by gamma counter correcting for radioactive decay using an aliquot of the injectate.

### Small-animal positron emission tomography

^89^Zr-αGPC3 imaging was performed using the Inveon PET/CT scanner (Siemens Medical Solutions USA, INC. Molecular Imaging, Knoxville, TN), which was calibrated for ^89^Zr. Tumor-bearing animals were imaged after injection with 11.1 MBq (300uCi) of ^89^Zr-αGPC3 (~ 70 μg antibody) via the tail vein. Four days after injection, whole-body PET and CT images were acquired on animals anesthetized with 1–2% isoflurane anesthesia in 100% oxygen at 1L/min in a temperature-controlled bed with respiratory monitoring. Animals first had a 60-min positron emission tomography scan followed by a computerized tomographic scan (~ 15 min), which enabled scatter and attenuation correction.

## Results

### ^89^Zr-aGPC3 accumulates in tumor with minimal off-target binding

**Fig. 1:**
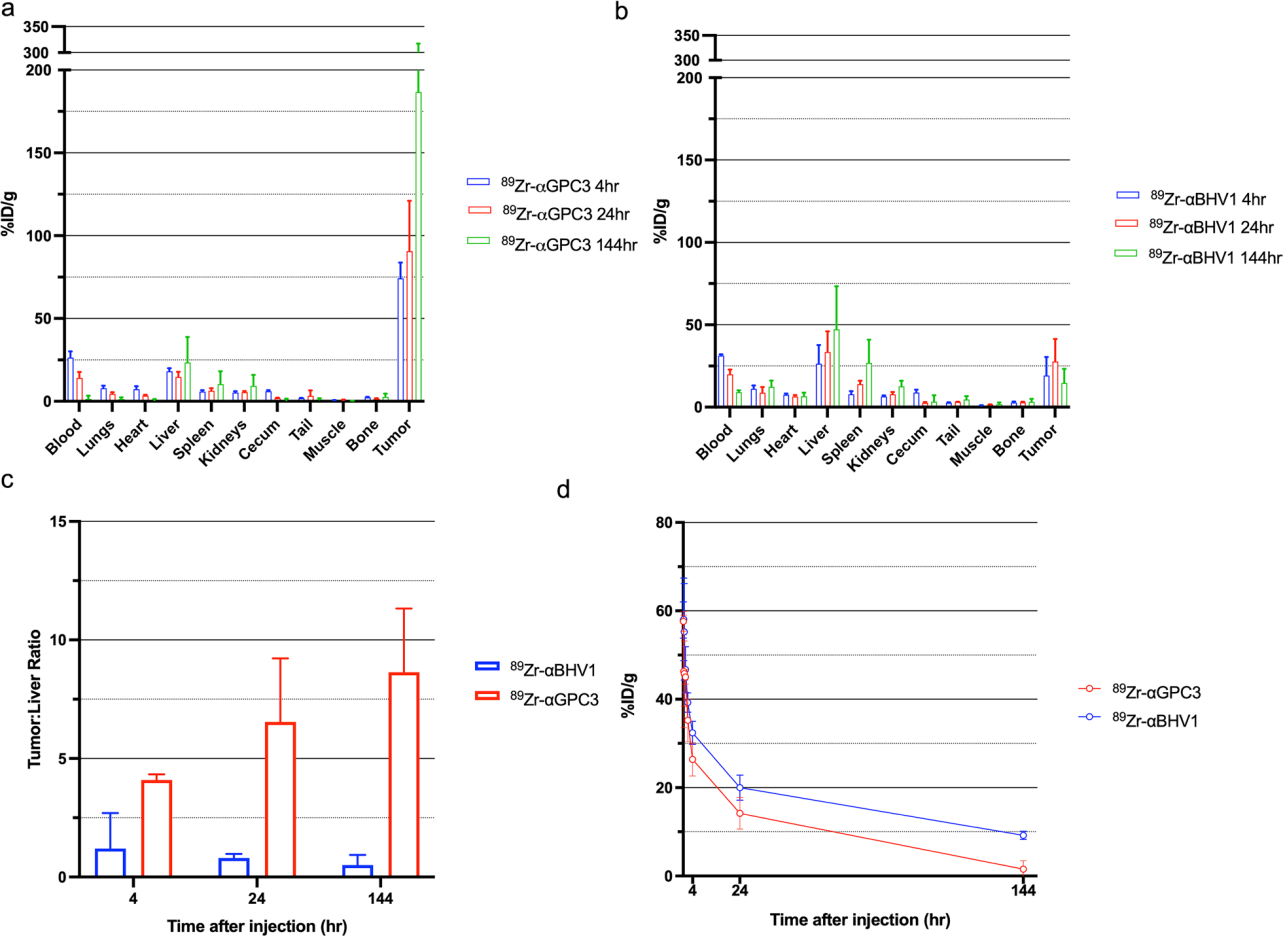
^89^Zr-aGPC3 avidly binds to tumor with minimal off-target binding. **a**, **b** Tissue biodistribution of ^89^Zr-aGPC3 and ^89^Zr-aBHV1 in tumor-bearing mice 4 h (n = 4), 24 h (n = 4), and 144 h (*n* = 3 for GPC3, *n* = 6 for BHV1) after injection. **c** Tumor-to-liver ratio 4 h, 24 h, and 144 h after injection. **d** Comparative blood clearance profiles in tumor-bearing mice at 5, 15, 30, 60, 120, 240 min, 24 h and 7 days after injection with of ^89^Zr-aGPC3 or ^89^Zr-aBHV1 (n = 4/time point). Bar and symbol denote mean, error bar denotes standard deviation

^89^Zr-aGPC3 accumulated in the tumor within four hours of injection with minimal off-target tissue uptake including the non-tumor liver and spleen (Fig. 1A). In contrast, the non-targeting control conjugate, ^89^Zr-aBHV1, did not significantly accumulate in the tumor and was sequestered in liver and splenic tissue (Fig. 1B). ^89^Zr-aGPC3 accumulated in the tumor over time with low non-tumor liver uptake. The tumor-liver ratio increased over 144 h due to increased tumor accumulation and non-tumor liver clearance (Fig. 1C). Pharmacokinetic analysis demonstrated that both ^89^Zr-aGPC3 and ^89^Zr-aBHV1 were cleared from the bloodstream over time, with ^89^Zr-aGPC3 cleared more rapidly likely due to increased tumoral uptake (Fig. 1D).

### GPC-3 targeted immuno-PET detects sub-centimeter tumors sensitively and specifically

**Fig. 2:**
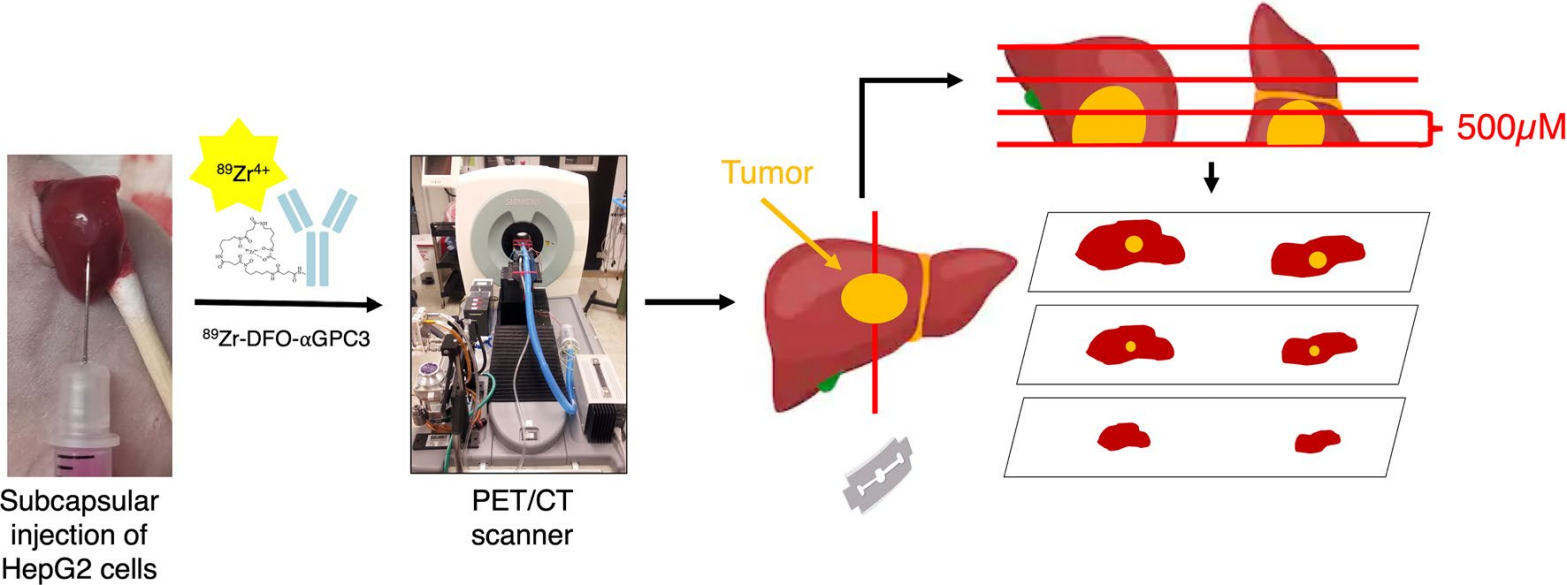
Experimental schematic. At pre-specified times after subcapsular hepatic injection of human HepG2 cells, 11.1 MBq of ^89^Zr-αGPC3 was injected via the tail vein. Four days after injection, whole-body PET and CT images were acquired. Animals were subsequently euthanized, and livers were assessed for tumors histologically

Forty-three animals were imaged at varying times after tumor implantation to assess the sensitivity and specificity of ^89^Zr-aGPC3 immuno-PET for a range of tumor sizes. The experimental schematic is shown in Fig. 2. After gross inspection and histologic processing, tumors were identified in 38 of 43 animals. Tumor diameters measured on histologic analysis ranged from 330 μm to 8.7 mm (median 2.6 mm, IQR 1.8).

**Fig. 3:**
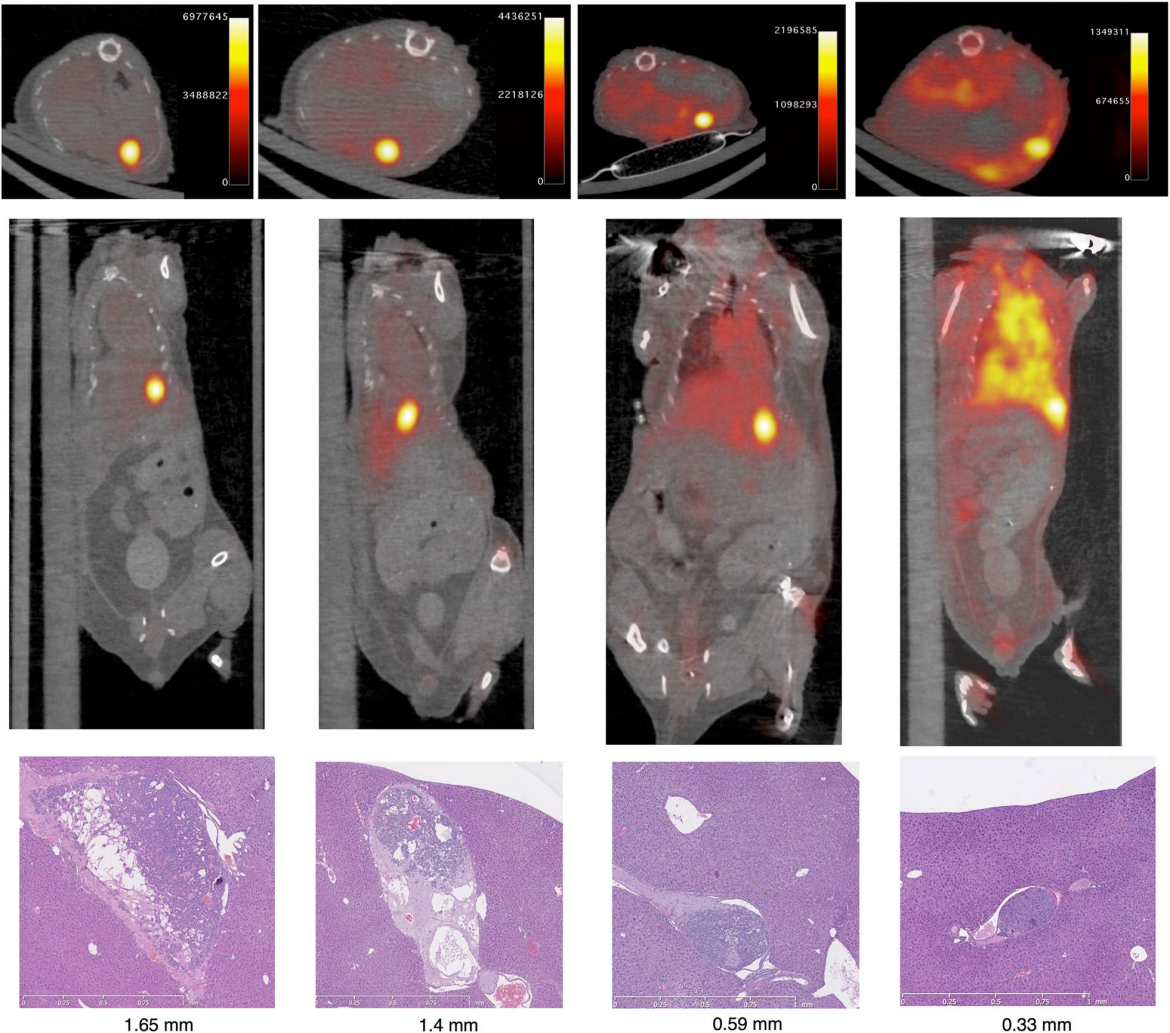
GPC-3 targeted immuno-PET detects sub-centimeter tumors sensitively and specifically. Representative axial (top) and coronal (middle) PET/CT-fused images of tumor-bearing animals 4 days after injection with ^89^Zr-aGPC3. Hematoxylin and eosin-stained hepatic tissue containing tumor corresponding to ^89^Zr-aGPC3 avid tumor (bottom). Maximum histologic cross-sectional diameter listed below

The sensitivity of ^89^Zr-aGPC3 immuno-PET was 100% (Table 1). Six tumors were not visible on gross inspection but were identified on histologic analysis and on PET imaging. Seven tumors identified were under 1 mm in diameter on histology, including one measuring 330 μm. Figure 3 displays representative axial and coronal PET/CT fused images of tumors detected with corresponding histologic images. PET images demonstrated high tumor-to-liver ratios with low background liver signal. In the smallest of tumors, the background liver and lung signals were higher due to lower radionuclide uptake and PET signal intensity in the tumor.

**Table 1:** Sensitivity and specificity of GPC3-targeted immuno-PET

	Histologic assessment	
	Positive	Negative
Immuno-PET		
Positive	38	2
Negative	0	3

Sensitivity and specificity of GPC3-targeted immuno-PET compared to histologic assessment

The specificity of ^89^Zr-aGPC3 was 60%. Two tumors identified on PET were not identified on histologic processing, representing false positive findings. These two tumors were minuscule on PET imaging and may have been missed on histologic sampling which was taken at 500 μm intervals. Three animals did not have tumors identified on PET or histologic analysis, representing true negative findings.

## Discussion

New diagnostic modalities are needed for sensitive and specific diagnosis of early HCC to reduce EIR and to identify antigens for targeted therapy. In this study,^89^Zr-aGPC3 had favorable in vivo biodistribution and pharmacokinetics, high sensitivity, and specificity for small GPC3^+^ tumors in an orthotopic xenograft model of HCC.

In a large cohort of 38 tumor-bearing animals, ^89^Zr-aGPC3 was highly sensitive, detecting 100% of tumors, including seven tumors under 1 mm in diameter as measured during histologic analysis. ^89^Zr-aGPC3 is highly avid for GPC3 [20, 21], and demonstrated high avidity for the tumor in vivo with minimal surrounding uptake in the normal liver, affording spatially resolute images for easy identification of small tumors. There were two falsely positive animals in the cohort with small tumors identified on PET imaging but not on histologic analysis. With only 5 non-tumor bearing animals in the cohort, these false positives yielded a low specificity of 60% in a small sample size. These tumors were diminutive on PET imaging and may have been missed during histologic processing.

Receptor-targeted molecular imaging and treatment hold promise for management of solid malignancies [10]. For example, galium-68 or copper-64 DOTATE PET/CT is a highly accurate modality for detection of neuroendocrine tumors through targeting of the somatostatin receptor and can detect sub-centimeter tumors missed by conventional CT or MR imaging [22]. Furthermore, it confirms the presence of the receptor targetable with therapeutic radionuclides like lutetium-177 [23]. For HCC, several novel molecular targets are being evaluated for early detection, prognostication, and therapy including GPC3, prostate specific membrane antigen (PSMA), CD146, and CD38 [12–17, 24]. GPC3 is currently a target for T cell therapies [25, 26], mAb antagonist and vaccine therapy [27, 28], and alpha and beta-particle radionuclide therapy [21, 29, 30]. GPC3-targeted immuno-PET may serve as a companion diagnostic to select patients for GPC3-targeted therapies.

A limitation of targeted technologies is the expression characteristics of tumor-associated antigens. Fortunately for HCC, GPC3 is highly expressed on the cell surface of HCC while being minimally expressed in cirrhotic or normal liver. However, most preclinical models rely on high GPC3-expressing cell lines which do not recapitulate HCC in situ. The use of human cell line in a mouse background favors high tumor uptake and minimal off-target binding with resulting high liver-to-tumor ratios and resolute PET imaging. Similarly, the imaging was performed in a small animal, with reduced partial volume and radiotracer attenuation effects, leading to high-resolution imaging unlikely to be replicated in human. The completeness of our histologic processing was limited by resources and by sectioning every 500 μm, potentially leading to a falsely low specificity.

## Conclusion

In conclusion, in a cell-line xenograft model of HCC, we observed highly specific binding of ^89^Zr-aGPC3 with high sensitivity and specificity for detection of sub-centimeter GPC3^+^tumors. ^89^Zr-aGPC3 may serve as a sensitive companion diagnostic for GPC3^+^ HCC.

## Supplementary Information

**Additional file 1.** Supplementary Methods.

## Data Availability

The datasets used and/or analyzed during the current study are available from the corresponding author on reasonable request.

## References

[1] Global Burden of Disease Liver Cancer C, Akinyemiju T, Abera S, Ahmed M, Alam N, Alemayohu MA, et al. The Burden of primary liver cancer and underlying etiologies from 1990 to 2015 at the Global, Regional, and National level: results from the global Burden of Disease Study 2015. JAMA Oncol. 2017;3(12):1683–91. 10.1001/jamaoncol.2017.3055 PMC5824275 28983565

[2] Marrero JA, Kulik LM, Sirlin CB, Zhu AX, Finn RS, Abecassis MM (2018). Diagnosis, staging, and management of hepatocellular carcinoma: 2018 practice guidance by the American association for the study of liver diseases. Hepatology.

[3] Cha C, Fong Y, Jarnagin WR, Blumgart LH, Dematteo RP (2003). Predictors and patterns of recurrence after resection of hepatocellular carcinoma. J Am Coll Surgeons.

[4] Poon RT, Fan ST, Lo CM, Liu CL, Wong J (1999). Intrahepatic recurrence after curative resection of hepatocellular carcinoma: long-term results of treatment and prognostic factors. Ann Surg.

[5] Tabrizian P, Jibara G, Shrager B, Schwartz M, Roayaie S (2015). Recurrence of hepatocellular cancer after resection: patterns, treatments, and prognosis. Ann Surg.

[6] Jelic S, Sotiropoulos GC (2010). Hepatocellular carcinoma: ESMO Clinical Practice Guidelines for diagnosis, treatment and follow-up. Ann Oncol.

[7] Baffy G (2015). Decoding multifocal hepatocellular carcinoma: an opportune pursuit. Hepatobiliary Surg Nutr.

[8] Lee YJ, Lee JM, Lee JS, Lee HY, Park BH, Kim YH (2015). Hepatocellular carcinoma: diagnostic performance of multidetector CT and MR imaging-a systematic review and meta-analysis. Radiology.

[9] Chernyak V, Fowler KJ, Kamaya A, Kielar AZ, Elsayes KM, Bashir MR (2018). Liver imaging reporting and data system (LI-RADS) version 2018: imaging of hepatocellular carcinoma in at-risk patients. Radiology.

[10] Ko YJ, Kim WJ, Kim K, Kwon IC (2019). Advances in the strategies for designing receptor-targeted molecular imaging probes for cancer research. J Control Release.

[11] Wei W, Rosenkrans ZT, Liu J, Huang G, Luo Q-Y, Cai W (2020). ImmunoPET: concept, design, and applications. Chem Rev.

[12] Hernandez R, Sun H, England CG, Valdovinos HF, Ehlerding EB, Barnhart TE (2016). CD146-targeted immunoPET and NIRF imaging of hepatocellular carcinoma with a dual-labeled monoclonal antibody. Theranostics.

[13] Sham JG, Kievit FM, Grierson JR, Miyaoka RS, Yeh MM, Zhang M (2014). Glypican-3-targeted 89Zr PET imaging of hepatocellular carcinoma. J Nucl Med.

[14] Sham JG, Kievit FM, Grierson JR, Chiarelli PA, Miyaoka RS, Zhang M (2014). Glypican-3-targeting F(ab')2 for 89Zr PET of hepatocellular carcinoma. J Nucl Med.

[15] Li S, England CG, Ehlerding EB, Kutyreff CJ, Engle JW, Jiang D (2019). ImmunoPET imaging of CD38 expression in hepatocellular carcinoma using (64)Cu-labeled daratumumab. Am J Transl Res.

[16] An S, Zhang D, Zhang Y, Wang C, Shi L, Wei W, et al. GPC3-targeted immunoPET imaging of hepatocellular carcinomas. Eur J Nucl Med Mol I 2022;49:2682–2692 10.1007/s00259-022-05723-x 35147737

[17] Yang X, Liu H, Sun CK, Natarajan A, Hu X, Wang X (2014). Imaging of hepatocellular carcinoma patient-derived xenografts using 89Zr-labeled anti-glypican-3 monoclonal antibody. Biomaterials.

[18] Baumhoer D, Tornillo L, Stadlmann S, Roncalli M, Diamantis EK, Terracciano LM (2008). Glypican 3 expression in human nonneoplastic, preneoplastic, and neoplastic tissues: a tissue microarray analysis of 4,387 tissue samples. Am J Clin Pathol.

[19] Guo M, Zhang H, Zheng J, Liu Y (2020). Glypican-3: a new target for diagnosis and treatment of hepatocellular carcinoma. J Cancer.

[20] Labadie K, Ludwig A, Lehnert A, Hamlin D, Kenoyer A, Daniel S (2020). GPC3 targeted theranostic platform for hepatocellular carcinoma. J Nucl Med.

[21] Ludwig AD, Labadie KP, Seo YD, Hamlin DK, Nguyen HM, Mahadev VM (2019). Yttrium-90-labeled anti-glypican 3 radioimmunotherapy halts tumor growth in an orthotopic xenograft model of hepatocellular carcinoma. J Oncol.

[22] Hofman MS, Lau WF, Hicks RJ (2015). Somatostatin receptor imaging with 68Ga DOTATATE PET/CT: clinical utility, normal patterns, pearls, and pitfalls in interpretation. Radiographics.

[23] Strosberg J, El-Haddad G, Wolin E, Hendifar A, Yao J, Chasen B (2017). Phase 3 trial of 177 Lu-dotatate for midgut neuroendocrine tumors. New Engl J Med.

[24] Kesler M, Levine C, Hershkovitz D, Mishani E, Menachem Y, Lerman H (2019). (68)Ga-PSMA is a novel PET-CT tracer for imaging of hepatocellular carcinoma: a prospective pilot study. J Nucl Med.

[25] Ishiguro T, Sano Y, Komatsu S-i, Kamata-Sakurai M, Kaneko A, Kinoshita Y, et al. An anti–glypican 3/CD3 bispecific T cell–redirecting antibody for treatment of solid tumors. Sci Transl Med. 2017;9(410):eaal4291. 10.1126/scitranslmed.aal4291 28978751

[26] Shi D, Shi Y, Kaseb AO, Qi X, Zhang Y, Chi J (2020). Chimeric antigen receptor-glypican-3 T-cell therapy for advanced hepatocellular carcinoma: results of phase I trials. Clin Cancer Res.

[27] Zhu AX, Gold PJ, El-Khoueiry AB, Abrams TA, Morikawa H, Ohishi N (2013). First-in-man phase I study of GC33, a novel recombinant humanized antibody against glypican-3, in patients with advanced hepatocellular carcinoma. Clin Cancer Res.

[28] Sawada Y, Yoshikawa T, Nobuoka D, Shirakawa H, Kuronuma T, Motomura Y (2012). Phase I trial of a glypican-3-derived peptide vaccine for advanced hepatocellular carcinoma: immunologic evidence and potential for improving overall survival. Clin Cancer Res.

[29] Bell MM, Gutsche NT, King AP, Baidoo KE, Kelada OJ, Choyke PL (2020). Glypican-3-Targeted Alpha Particle Therapy for Hepatocellular Carcinoma. Molecules.

[30] Labadie KP, Hamlin DK, Kenoyer A, Daniel SK, Utria AF, Ludwig AD (2021). Glypican-3 targeted thorium-227 alpha therapy reduces tumor burden in an orthotopic xenograft murine model of hepatocellular carcinoma. J Nucl Med.

