# Supplementary material for: Glypican-3 targeted positron emission tomography detects sub-centimeter tumors in a xenograft model of hepatocellular carcinoma
Source: EJNMMI Res. 2023 Apr 27;13:35. doi: 10.1186/s13550-023-00980-9 (PMC10140215; doi:10.1186/s13550-023-00980-9)
Supplement: Supplementary file 1 — Additional file 1. Supplementary Methods. [file 13550_2023_980_MOESM1_ESM.docx]

Supplemental Methods

**Orthotopic, cell-line xenograft model development**

HepG2-Red-FLuc (HepG2) cells expressing GPC-3 and *Luciferase* from PerkinElmer (Bioware, cat. no. BW134280, RRID:CVCL_5I98) were passaged fewer than 10 times after thawing for use in described experiments. We did not perform *Mycoplasma* testing. Detached HepG2 cells created a cell suspension, and we centrifuged the suspension at 1,250 rpm for 10 minutes at 4^o^C. We suspended the cell pellet in cooled Matrigel (BD Biosciences) to achieve final concentration of 10^8^ cells/mL.

After a week of acclimatizing in the animal facility, we anesthetized mice using 1.5% inhaled isoflurane and exposed the left lobe of the liver through a 0.7 cm upper midline laparotomy. We injected 20 μL Matrigel-cell suspension into the subcapsular space of the left hepatic lobe slowly to create a subcapsular wheal, taking care not to burst the wheal. We then closed the laparotomy in two layers using a simple interrupted technique with 4-0 braided synthetic suture followed by simple interrupted, buried technique with 5-0 monofilament synthetic suture.

**Histopathologic Processing and Cross-Sectional Diameter Measurement**

To collect livers for histopathologic analysis, we anesthetized the animals with inhaled 1.5% isoflurane, removed the livers en bloc and fixed them in 10% phosphate buffered formalin for four weeks. To assess for presence of tumors, we inspected the livers grossly. If tumor was present on inspection, we bisected the liver with a scalpel at the largest visible cross-sectional area of the tumor. If tumor was not present, we looked for an injection site scar in the left lobe for bisection. We oriented the bisected liver and embedded it in Histogel Specimen Processing Gel (ThermoScientific, Cat. #HG-4000-012) *en face*. We embedded the Histogel specimen in paraffin and then sectioned the entire liver at 500 μm intervals and placed on a positively charge slide. The University of Washington Histology and Imaging Core performed hematoxylin and eosin (H&E) staining on all slides. We used the Hamamatsu Nanozoomer Whole Slide scanner to image each liver section. We measured the greatest axial diameter on the section with the largest transverse area of tumor was using the NanoZoomer Digital Pathology imaging software (Hamamatsu).

**Conjugation of αGPC3 and *α*BHV1 with Deferoxamine (DFO)**

We demetallated the *α*GPC3 and *α*BHV1 ntibody for labeling with radiometals and concentrated the solution to 6mg/mL and dialyzed against metal-free saline (150mM NaCl, and 1 mM EDTA adjusted to pH 7 and passed over a Chelex 100 column) for 3 days at 4°C with a minimum of three buffer changes per day. One day prior to conjugation, we dialyzed the antibody for an additional two buffer changes to replace the saline with demetallated HEPES buffer (50mM HEPES (*N*-(2-hydroxyethyl)piperazine-*N*′-ethanesulfonic acid, 150mM NaCl, and 1mM EDTA adjusted to pH 8.5 and passed over a Chelex 100 column). We then conjugated the demetallated *α*GPC3 or *α*BHV1 antibody with 10 equivalents of p-SCN-Bn-DFO as a solution at 10mg/mL in DMSO in acid washed microcentrifuge tubes. These reactions ran overnight at room temperature with gentle mixing and were then dialyzed against a metal-free citrate buffer (50mM sodium citrate and 150mM NaCl with pH 5.5) over 3 days at 4°C followed by dialysis against 150mM saline (pH 7.0) for another 3 days. Each buffer change contained Chelex resin to scavenge metals. We stored the final conjugates in acid washed tubes at 4°C.

**^89^Zr-Labeling of αGPC3-DFO and *α*BHV1-DFO**

We added demetallated 2M sodium carbonate to ^89^Zr to adjust the pH to 7.0-7.5 and then added HEPES buffer at pH 7.0 to the ^89^Zr solution, followed by *α*GPC3-DFO or *α*BHV1, prepared as above. We incubated this mixture for 2 hours at room temperature and separated the labeled antibody from unreacted ^89^Zr via a PD-10 column (GE Healthcare) eluted with PBS prior to analysis by HPLC to verify radiochemical purity. We used acid-washed vials and pipette tips for all steps. he radiochemical yield for Zr89-DFO-GPC was 71.2% and Zr89-DFO-BHVI was 87.6%.

**PET/CT Image Reconstruction**

We reconstructed PET images using ordered subset expectation maximization/shifted Poisson maximum a posteriori (OSEM3D/SP-MAP; 2 iterations 18 subsets) with a 256x256 matrix, target resolution of 1.5mm, zoom factor 1.3, and corrections for scatter and attenuation. We reconstructed CT images using forward back projection with a Shepp-Logan filter, slight noise reduction, and appropriate beam hardening corrections.
